# Decreased Analgesic Requirements in Super Morbidly Versus Morbidly Obese Patients Undergoing Laparoscopic Sleeve Gastrectomy

**DOI:** 10.1007/s11695-020-04559-4

**Published:** 2020-04-03

**Authors:** Hamed Elgendy, Talha Youssef, Ahmad Banjar, Soha Elmorsy

**Affiliations:** 1 Department of Anaesthesia, Assiut University Hospitals, Assiut, Egypt; 2 Anaesthesia Dept., Al Wakrah Hospital, HAMAD Medical Corporation, P.O. Box 82228, Doha, Qatar; 3 Qatar University & Weill Cornel Medicine Qatar, Doha, Qatar; 4 Internal Medicine Department, Prince Mohammad Bin Abdul-Aziz Hospital, Ministry of National Guard, Al Madinah, Saudi Arabia; 5 Umm Al Qura University & King Abdullah Medical City, Internship Program, Makkah, Makkah, Saudi Arabia; 6 Medical Pharmacology, Faculty of Medicine, Cairo University, Giza, Egypt

**Keywords:** Morphine, Pain, Multimodal analgesia, Bariatric surgery, Obesity

## Abstract

**Background:**

Scarce data exists about analgesic requirements in super morbidly obese (SMO) patients who underwent sleeve gastrectomy. We attempted to investigate analgesic requirements for SMO, when compared with morbidly obese (MO) individuals who underwent sleeve gastrectomy and its impact on postoperative outcome.

**Methods:**

We studied 279 consecutive patients (183 MO, 96 SMO) who underwent bariatric surgery. Data analysis included perioperative anaesthetic management, analgesic consumptions, opioids side effects, and ICU admission.

**Results:**

The SMO group showed higher patients with asthma, epilepsy, obstructive sleep apnoea (OSA), and ASA III percentages (*P =* 0.014, *P =* 0.016, *P ˂* 0.001, and *P ˂* 0.001, respectively). There were no significant differences in the total morphine consumption intraoperatively, or after 24 h. However, reduced consumption of intraoperative fentanyl and morphine in SMO when calculated per total body weight (TBW) (*P =* 0.004 and *P =* 0.001, respectively). At PACU, tramadol consumption per TBW and lean body mass (LBM) were significantly reduced in SMO (*P =* 0.001 and *P =* 0.025, respectively). Paracetamol consumption was significantly reduced in the SMO group (*P =* 0.04). They showed higher comorbidities (*P ˂* 0.001), longer anaesthesia time (*P =* 0.033), and greater ICU admissions (*P ˂* 0.001). Vomiting was higher in the MO group (*P =* 0.004). Both groups showed comparable pain scores (*P =* 0.558) and PACU stay time (*P =* 0.060).

**Conclusions:**

Super morbidly obese patients required fewer opioids and analgesics perioperatively. They exhibited higher comorbidities with greater anaesthesia time and ICU admissions. PACU stay time and pain scores were comparable.

## Introduction

High-risk super morbidly obese patients are considered a challenge for the anesthesiologist who required tailored anaesthetics management especially opioid components to avoid hypoxemia and hypoventilation [1]. Supplementation of opioids has been associated with abnormal breathing patterns such as obstructive sleep apnoea (OSA) and hypoxemia as well as obstruction of the upper airway [2, 3]. Doufas and his colleagues [4] explained that nocturnal hypoxemia in individuals at high risk for OSA was directly associated with increasing potency of opioid analgesics. Super morbidly obese patients who underwent laparoscopic surgery were more prone to have associated morbidities and challenges intraoperatively, not only difficult intubation and ventilation aspects but also surgical difficulties and complications [5].

Sultana et al. recommended the use of opioid-free analgesia and optimal multimodal analgesia to prevent complications in compromised patients [6].

Moreover, increasing individuals with opioid use disorder in the USA attract attention to the perioperative period which may initiate the new persistent opioids use [7]. Unused oral opioid prescription after surgery can initiate dependence and abuse [8]. In our institute, we have a conservative opioid protocol, with tailored perioperative opioids and implementations of multimodal analgesia without postoperative oral opioid prescriptions.

Pain after bariatric surgery may be significant and harms patients’ emergence, postoperative respiration functions, hemodynamic parameters, and incidence of postoperative nausea and vomiting (PONV). Moreover, delayed ambulation, ICU admission, and mortality can also be a consequence [9, 10].

Few studies of postoperative pain management in bariatric surgery have been reported [11, 12]. However, no studies are investigating super morbidly obese patients’ analgesic requirements.

### Aim of This Study

The primary outcome was to examine the variation in analgesic needs of morbidly and super morbidly obese patients. The secondary outcome was to follow up postoperative outcomes of those patients regarding PONV, pain management, post-anaesthesia care unit (PACU) stay time, and ICU admission.

## Methods

This retrospective study was approved and registered by our institutional review board committee of King Abdullah Medical City, Makkah, Saudi Arabia (clinical trial registration no. 14-146). Perioperative patient outcome data were collected and recorded from January 2015 to January 2017. Obesity is defined as a body mass index (BMI) more than 30 kg/m^2^, and morbid obesity defined as (BMI 40–49.9 kg/m^2^) and super morbid obesity (BMI ≥ 50–69.9 kg/m^2^) [13]. Patients were assigned according to their BMI into morbidly obese (MO), group I, and super morbidly obese (SMO), group II. Consecutive patients (183, MO group; 96, SMO group) who have undergone laparoscopic sleeve gastrectomy under general anaesthesia were included.

Sleeve gastrectomy was performed by two experienced surgeons, who are contemporaries and use similar surgical techniques. All patients were evaluated prior to the surgical procedure approximately 15 days in the pre-anaesthetic assessment clinic. In the holding area, a second pre-operative evaluation was performed for potential difficulty in airway management.

A Mallampati score of 3 or 4, neck circumference more than 40 cm, a thyromental distance less than 6.5 cm, and large tongue or limitations in cervical or mandibular mobility were each considered a predictor for difficult intubation. Our intubation strategy, if only neck circumference more than 40 cm, but there were no other predictors for difficult intubation, we proceed using an optimized blade size of a regular laryngoscope. But, if there were 1–2 predictors of the previous criteria, we optimized video-assisted intubation method or fiberoptic intubation according to the possible expected airway difficulty.

General data were collected including patients’ gender, age, BMI, ASA physical status, preoperative laboratory parameters, associated medical comorbidities, their medications, perioperative monitoring, anaesthesia and surgery duration, perioperative complications, PACU duration, and intensive care unit admission. Specific perioperative data collected included intra-operative premedication, IV anaesthetic dosages, muscle relaxants, anaesthetic adjuvants, and analgesics. Difficult airway assessment, Cormack-Lehane grade, intubation methods as well as total fluid intake, blood loss, and urine output data were collected.

Routine intraoperative monitoring included lead II and V, ECG, non-invasive blood pressure or invasive blood pressure in the case of indicated patients, pulse oximetry, end-tidal CO_2_ (EtCO_2_), body temperature, and neuromuscular monitor using acceleromyography of the adductor pollicis muscle (Train of four, TOF-Guard). Bispectral index (BIS) monitoring (BIS monitor model A 2000,) was routinely used. In subjects with severe cardiac comorbidities or with great technical difficulties in peripheral venous access, central venous pressure catheter (CVP) was inserted.

Balanced general anaesthesia, including prophylactic antiemetic therapy, was provided by three experienced anaesthesiologists. Supplemental intra-operative analgesia was administered based on the clinical decision and our institution protocol.

An induction dose of propofol (1–2.5 mg/kg) according to the lean body weight (LBW) (Diprifusor, Fresenius, Schelle, Belgium) and an intravenous (IV) administration of fentanyl (1–2 μ/kg) were scaled and keep hemodynamics within 15% of preoperative values and tracheal intubation was facilitated using rocuronium bromide (1 mg/kg) IV or (0.4 mg/kg) for atracurium besylate. Some patients expected difficult intubation; their intubation was performed using succinylcholine in a dose of 1.5 mg/kg and later, non-depolarizing muscle was given. Reversal of muscle relaxant was done using neostigmine methylsulfate (0.05–0.07 mg/kg) body weight, and glycopyrrolate (0.4 mg) IV. Ranitidine (150 mg) IV was given for gastric protection in addition to (4 mg) ondansetron IV and dexamethasone (8 mg) IV for nausea and vomiting prophylaxis. Our anesthesiologists chose either sevoflurane or desflurane as inhalation maintenance anaesthetics. A 5000 IU subcutaneous heparin was used in all the patients for venous thromboprophylaxis.

We calculated the amounts of analgesics consumed by our patients as per kilogram body weight and per lean body mass. Lean body mass (LBM) is a part of body composition that is defined as the difference between the total body weight and body fat weight.

Generally, men have a higher proportion of LBM than women do. The dosages of some anaesthetic agents, particularly water-soluble drugs, are routinely based on the LBM. Multiple formulas have been developed for calculating estimated LBM (eLBM) and the calculation above provides the results for all of them. Lean body mass formula for adults, the Hume formula [14], is as follows:

For males: eLBM = 0.32810 W + 0.33929H − 29.5336

For females: eLBM = 0.29569 W + 0.41813H − 43.2933

In the formulas above, *W* is the body weight in kilogram and *H* is the body height in centimeter.

Intra-operative paracetamol was given as 1 g IV infusion, maximum 4 times/24 h. In post-anaesthesia care unit (PACU), analgesia consisted of fentanyl increments. IV tramadol, paracetamol, and lornoxicam were also administered when required and recorded.

Total morphine consumption equivalent was calculated for intra operative fentanyl and morphine together as described before by the American pain society [15].

Pain was categorized based on a numeric visual analogue scale (VAS) (0 = no pain and 10 = severe pain). Hemodynamics such as systolic blood pressure (SBP), diastolic blood pressure (DBP), and heart rate (HR) were recorded.

Postoperative variables such as VAS on PACU entry and discharge, postoperative nausea/vomiting (PONV) and medications used to manage were recorded. In the surgical ward, analgesia protocol, morphine 5 mg, subcutaneous/6 h, PRN (when needed); IV paracetamol 1 g/6 h, PRN; and tramadol 100 mg, IV/8 h, PRN were recorded. Opioid analgesia and other analgesic drugs required in the first 24 h were recorded. On the floor, continuous O_2_ saturation monitoring was administered for every bariatric patient. ICU admission was based on an elective manner based on their perioperative assessment and risk profile. Other exceptional unplanned ICU admissions were recorded. Data were analysed using SPSS® version 21 (IBM Corp., Armonk, NY, USA) and included descriptive statistics. Bivariate analysis was performed using Student’s *t* test. Differences between groups were compared using Pearson’s chi-square test or Fisher’s exact test as appropriate. *P* value < 0.05 was considered statistically significant.

## Results

Two hundred and seventy-nine patients were enrolled in the study,183 in group I, MO, and 96 in group II, SMO. Both groups were similar regarding demographics except for group II which had higher patients with asthma, epilepsy, obstructive sleep apnoea (OSA), and ASA III percentages, (*P =* 0.016, *P =* 0.014, *P ˂* 0.001, and *P ˂* 0.001, respectively) as shown in Table.1. Preoperative laboratory parameters were comparable except low serum albumin (3.6 ± 0.8) and high INR (1.01 ± 0.12) in the SMO group (*P ˂* 0.001, *P =* 0.020, respectively). We revised liver functions of the two groups; they were comparable as the following readings: total bilirubin mg/dL, 0.4 ± 0.3 (MO), 0.5 ± 0.3 (SMO), (*P =* 0.208); ALT (IU/L), 46.2 ± 13.2 (MO), 47.6 ± 25.5 (SMO), (*P =* 0.928); AST (IU/L), 24.9 ± 13.2 (MO), 26.3 ± 18.8 (SMO), (*P =* 0.694), respectively.

**Table 1:** Demographic characteristics

Characteristics	Morbidly obese, *n* = 183	Super morbidly obese, *n* = 96	*P* value
Age (years)	34.5 ± 10.4	34.1 ± 10.7	0.753
Gender, *n* (%)			
Male	72/183 (39.3)	46/96 (47.9)	0.169
Female	111/183 (60.7)	50/ 96 (52.1)	0.169
Weight (kg)	118.8 ± 18.0	151.6 ± 23.1	˂ 0.001
Height (m)	1.7 ± 0.1	1.6 ± 0.1	0.005
Mallampati classification, *n* (%)			
I	40/183 (21.9)	24/96 (25.0)	0.835
II	114/183 (62.3)	57/96 (59.4)	
III	29/183 (15.8)	15/96 (15.6)	
ASA classification, *n* (%)			
Class II	39/183 (21.3)	0/96 (0.0)	˂ 0.001
Class III	144/183 (78.7)	96/96 (100.0)	˂ 0.001
Comorbidities, *n* (%)			
HTN			
No	147/ 183 (80.3)	76 /96 (79.2)	0.818
Yes	36/183 (19.7)	20/96 (20.8)	
DM			
No	149/183 (81.4)	77/96 (80.2)	0.806
Yes	34/183 (18.6)	19/96 (19.8)	
Renal			
No	178/183 (97.3)	96/96 (100.0)	0.102
Yes	5/183 (2.7)	0/96 (0.0)	
CNS			0.016
No	183/183 (100.0)	3/96 (3.1)	
Yes	0/183 (0.0)	3/96 (3.1)	
Bronchial asthma			
No	176/183 (96.2)	85/96 (88.5)	0.014
Yes	7/183 (3.8)	11/96 (11.5)	
Obstructive sleep apnoea			
No	180/183 (98.4)	83/96 (86.5)	˂ 0.001
Yes	03 /183 (1.6)	13/96 (13.5)	
Baseline SBP (mmHg)	119.17 ± 18.82	118.01 ± 18.33	0.622
Baseline DBP (mmHg)	74.84 ± 11.01	74.34 ± 10.77	0.712
Baseline HR (bpm)	83.75 ± 14.10	84.27 ± 12.48	0.764

Values are means ± SD and number, percentage

*P* ˂ 0.05 considered statistically significant

*MO*, morbidly obese; *SMO*, super morbidly obese; *ASA*, the ASA physical status classification system is a system for assessing the fitness of patients before surgery; Mallampati Classification: *Class 0*, ability to see any part of the epiglottis upon mouth opening and tongue protrusion; *Class I*, soft palate, fauces, uvula, pillars visible; *Class II*, soft palate, fauces, uvula visible; *Class III*, soft palate, base of uvula visible; *Class IV*, soft palate not visible at all; *SBP*, systolic blood pressure; *DBP*, diastolic blood pressure; *HR*, heart rate

There were no significant differences in total morphine consumption intra-operatively or at first 24 h postoperatively. Also, comparable VAS scores were seen in both groups either at PACU entry or PACU discharge (Table 2). However, when accounting for intra-operative fentanyl and end of surgery morphine given, calculated per total body weight (TBW) and lean body mass (LBM) revealed the following: reduced consumption of fentanyl per TBW and morphine in group II significantly (*P =* 0.004 and *P =* 0.001, respectively). Despite the fact that values per LBM are insignificant statistically, they reduced in group II when compared with those in group I.

**Table 3:** Intra-operative variables

Variable	Morbidly obese, *n* = 183	Super morbidly obese, *n* = 96	*P* value
Premedication: midazolam (mg)	0.3 ± 0.9	0.2 ± 0.6	0.508
Difficult Intubation, *n* (%)	08/183 (4.4)	08/96 (8.3)	0.176
Cormack-Lehane grade			
Grade I	93/183 (50.8)	44/96 (45.8)	0.639
Grade II	74/183 (40.4)	46/96 (47.9)	
Grade III	14/183 (7.7)	05/96 (5.2)	
Grade IV	02/183 (1.1)	01/96 (1.0)	
Airway intubation method, *n* (%)			
Regular laryngoscope	138/183 (75.4)	57/96 (59.4)	0.033
Video -assisted	37/183 (20.2)	29/96 (30.2)	
Fibre-optic intubation	05/183 (2.7)	06/96 (6.3)	
Retro molar device	03/183 (1.6)	04/96 (4.2)	
Total crystalloids (mL)	2293.7 ± 778.1	2694.8 ± 835.8	˂ 0.001
Total blood loss (mL)	19.8 ± 97.4	59.0 ± 321.2	0.367
Total urine output (mL)	103.4 ± 272.9	190.1 ± 385.2	0.017
Total anaesthesia time (min)	127.7 ± 38.1	137.6 ± 42.6	0.033
Total surgery time (min)	102.7 ± 35.1	107.2 ± 39.3	0.366

Values are either mean ± SD or number and percentage. *P* ˂ 0.05 considered statistically significant

Cormack and Lehane, Grade 1: most of the glottic opening can be seen with. Grade 2, only the posterior portion of the glottis or only arytenoid cartilages are visible. Grade 3, only the epiglottis but no portion of the glottis is visible, whereas in Grade 4, neither the glottis nor the epiglottis can be see

**Table 2:** Analgesic usage and pain managements

Variables	Morbidly obese, *n* = 183	Super morbidly obese, *n* = 96	*P* value
VAS at PACU entry	2.59 ± 1.62	2.43 ± 1.53	0.558
VAS at PACU discharge	1.86 ± 1.07	1.70 ± 1.02	0.176
Total morphine equivalent intra operative and PACU	10.5 ± 4.5	10.6 ± 4.4	0.878
Total morphine equivalent at first 24 h	22.6 ± 9.6	23.8 ± 8.7	0.76
Intra operative fentanyl consumption (μ/kg)	1.67 ± 0.75	1.43 ± 0.63	0.004
Intra operative fentanyl consumption (μ/LBM)	3.19 ± 1.40	3.05 ± 1.39	0.409
End of surgery morphine consumption (mg/kg)	0.08 ± 0.02	0.06 ± 0.02	0.001
End of surgery morphine consumption (mg/LBM)	0.71 ± 0.72	0.15 ± 0.14	0.313
PACU tramadol consumption (mg/kg)	0.7479 ± 0.245	0.5660 ± 0.171	0.001
PACU tramadol consumption (mg/LBM)	1.4073 ± 0.476	1.1598 ± 0.345	0.025
PACU Lornoxicam consumption (mg/kg)	0.0966 ± 0.038	0.0878 ± 0.029	0.527
PACU Lornoxicam consumption (mg/LBM)	0.1905 ± 0.062	0.1797 ± 0.064	0.689
Total paracetamol consumption (g/kg)	0.0176 ± 0.003	0.0153 ± 0.004	0.040
Total paracetamol consumption (g/LBM)	0.0331 ± 0.005	0.0326 ± 0.008	0.844

NOTE: total morphine equivalent intra-operative fentanyl, calculated as explained in “Methods” section

Values are means ± SD. *P* ˂ 0.05 considered statistically significant

*PACU*, post-anaesthesia care unit; *VAS*, visual analogue score; *IV*, intravenous; *ICU*, intensive care unit; *LBM*, lean body mass

**Table 4:** Postoperative outcome variables

Variable	Morbidly obese, *n* = 183	Super morbidly obese, *n* = 96	*P* value
PACU stay time (min)	90.4 ± 41.0	97.3 ± 31.9	0.060
Nausea			
No	154 /183 (82.2)	83/96 (86.5)	0.609
Yes	29 /183 (15.8)	13/96 (13.5)	
Vomiting			
No	165 /183 (90.2)	93/96 (96.9)	0.044
Yes	18 /183 (9.8)	3/96 (3.1)	
PACU discharge SBP (mmHg)	129.65 ± 17.88	129.98 ± 14.80	0.880
PACU discharge DBP (mmHg)	71.37 ± 11.20	69.17 ± 11.42	0.153
PACU discharge HR (bpm)	75.62 ± 13.01	73.41 ± 12.62	0.103
ICU admission, *n* (%)			
No	180 /183 (98.4)	84/96 (87.5)	˂ 0.001
Yes	03 /183 (1.6)	12/96 (12.5)	

Values are either mean ± SD or number and percentage. *P* ˂ 0.05 considered statistically significant

*PACU*, post-anaesthesia care unit; *ICU*, intensive care unit; *SBP*, systolic blood pressure; *DBP*, diastolic blood pressure; HR, heart rate

Other analgesic consumptions at PACU, tramadol consumption per kilogram and per LBM was significantly reduced in group II (*P =* 0.001 and *P =* 0.025, respectively). Total paracetamol consumption intra-operatively and at PACU was significantly reduced in group II (*P =* 0.04), as shown in Table 2.

Difficult intubation event percentage and grading percentage according to Cormack-Lehane were comparable respectively (*P =* 0.176, *P =* 0.639) in both groups. Regarding the intubation method, the regular laryngoscope constituted a higher percentage in the MO versus SMO group, while other methods such as video-assisted laryngoscopy showed a higher percentage in the SMO group when compared with MO (*P =* 0.003). Anaesthesia time, total crystalloid intake, and urine output were higher in the SMO group versus the MO group, respectively (*P =* 0.033, *P ˂* 0.001, *P =* 0.017), as shown in Table 3. There were insignificant differences between the two groups regarding muscle relaxant, their reversal, and anaesthetic gases. Stable haemodynamic parameters were exhibited in baseline and PACU values for both groups as shown in Tables 1 and 4.

Opioid side effects, only one case of group II, had delayed extubation, mostly naïve exposure to opioids, and kept intubated during transfer to ICU. There was increased vomiting in the MO group than in the SMO (*P =* 0.004).

Regarding the outcome, both groups had comparable PACU stay time (*P =* 0.060). However, elective ICU admission from the operative theatre was attributed to the severity of comorbidities which was higher in the SMO group (II), 12.5% (*n =* 12) than in the MO group (I), 1.6% (*n =* 3) (*P ˂* 0.001) as shown in Table 4. Mostly, OSA was the cause for ICU elective admission, except one case in group II which developed low oxygen saturation on the floor and therefore was transferred to ICU in an unplanned manner.

## Discussion

In this study, we found that super morbidly obese patients consumed lesser amounts of opioids and other analgesics according to their total body weight calculations when compared with morbidly obese individuals. However, the two groups had comparable total analgesics required on the first postoperative day. Group II exhibited more patients with comorbidities. Besides, they have increased anaesthesia time and ICU admissions without affecting their PACU stay time. The majority of MO cases showed easy intubation, while video-assisted laryngoscopy was used frequently in SMO, which exhibited more difficult intubation cases. Increased incidence of vomiting in MO patients may be attributed to relative higher opioid dosages when compared with the calculations per kilogram and per LBM consumption in super morbidly individuals.

Alteration of pharmacokinetics properties of opioids in obese individuals could be a result of increased cardiac output and changes in body composition such as increases in fat and lean mass. Cardiovascular and respiratory pathophysiological derangements make these patients more susceptible to opioid-induced respiratory depression and airway obstruction [16, 17]. Abnormal preoperative laboratory tests, such as low albumin [18] and high INR [19], were reported before in obese patients; however, they did not influence their perioperative management.

Safe analgesic options, while providing optimal analgesic management, remain a challenge. Taylor et al. [20] reported that 77% of opioid-related morbidity occurred on the first postoperative day. According to the American Society of anaesthesiologist (ASA) database, approximately half of pulmonary events secondary to opioids were in morbidly obese subjects [21]. Doufas and his colleagues [4] explained that nocturnal hypoxemia in individuals at high risk for OSA was directly associated with increasing potency of opioid analgesics.

Obesity not only increases postoperative pulmonary consequences but also the higher affinity to opioids increases the incidence of these adverse events occurring during the perioperative management. This concern of opioid-related respiratory complications makes our analgesic administration to be tailored. Our institute has a conservative perioperative opioid protocol without postoperative oral opioid prescriptions. We are trying to avoid the nightmare of opioid dependence in North America which could be initiated during the perioperative course in such cardiopulmonary compromised individuals [7].

To achieve the desired pain relief, the alert anaesthesiologist resorts to a multimodal analgesic strategy with a significant degree of success, using fewer opioids, in addition to the use of anaesthesia adjuvants wherever feasible as we followed in our study. A combined approach of multiple analgesic modalities acting at different pain target pathways has shown promising results in obese patients with compromised airway [22]. Implementation of multimodal analgesia in our institute may be contributed to reduce opioid requirements either intraoperatively or postoperatively as reported in a previous study [23].

Endogenous opiates have been linked to pain regulation. It has also been found that higher endogenous opiate levels lower the pain sensitivity of an individual. Experimental studies have confirmed that basal beta-endorphin levels are higher in genetically engineered obese mice [24]. Consequently, it is alleged that obese patients are more likely to have higher basal endogenous opioid levels when compared with the non-obese population. This could explain our results that analgesic requirements in super obese were comparable with those in less obese subjects.

Inconsistent with a clinical study, Rand and his colleagues [25] demonstrated that obese patients undergoing abdominal surgeries required much fewer opioids for comparable levels of analgesia in contrast to the lean individuals undergoing similar surgeries.

Obese patients are also known to show an enormous degree of inter-individual variation in opioid requirements [26]. This could be attributed to the pathophysiological changes produced by obesity. It can markedly affect the distribution, binding, and elimination of opioids; thus, predicting a “low-safe” and yet therapeutically effective opioid dose in obese becomes extremely challenging. Therefore, not only morbidly obese patients are more sensitive to opioids but also they may require much less opioid doses to achieve similar analgesic endpoints [26].

Pre-emptive analgesia may be additionally used to improve the efficacy of postoperative pain relief while allowing further reductions in opioid requirements. Providing safe and adequate analgesia is an obligation for any anesthesiologist taking care of any surgical patient. He should tailor the balance between safety and efficacy [27, 28]. Further prospective studies in analgesic options are necessary to face this challenge.

Increased ICU admission for super obese subjects was expected as they have higher patients with asthma [29], epileptic comorbidities, and increased OSA which indicated the continuation of care and required elective admission except for few cases. It could be explained with our results which indicated comparable PACU time for both groups, as individuals who had planned ICU admission were transferred directly to ICU without staying in PACU.

This study has some limitations. We studied retrospectively those consecutive patients who were subjected to sleeve gastrectomy. A detailed prospective study with pharmacokinetic analysis for opioids requirements in super-obese patients and their endogenous opioid levels is warranted.

## Conclusions

Super morbidly obese patients consumed fewer opioids and analgesics perioperatively versus morbidly obese individuals. They have higher comorbidities, OSA, and longer anaesthesia time with greater ICU admissions. Both groups had comparable pain scores and hemodynamic stability.
